# Supplementary material for: The Role of Prussian Blue-Thallium and Potassium Similarities and Differences in Crystal Structures of Selected Cyanido Complexes of W, Fe and Mo
Source: Materials (Basel). 2022 Jun 29;15(13):4586. doi: 10.3390/ma15134586 (PMC9267926; doi:10.3390/ma15134586)
Supplement: Supplementary file 1 [file materials-15-04586-s001.zip › materials-1792384-Supplementary.pdf]

Supplementary Information

How Prussian Blue can act? Thallium and potassium similarities and differences in crystal structures of selected cyanido complexes of W, Fe and Mo

Maciej Hodorowicz,[1,2*] Janusz Szklarzewicz[1] and Anna Jurowska[1]

[1] Jagiellonian University, Faculty of Chemistry, Gronostajowa 2, 30-387 Kraków, Poland

[2] Institute of Chemistry, Jan Kochanowski University in Kielce, 7 Uniwersytecka Str., 25-406 Kielce, Poland

IR spectra

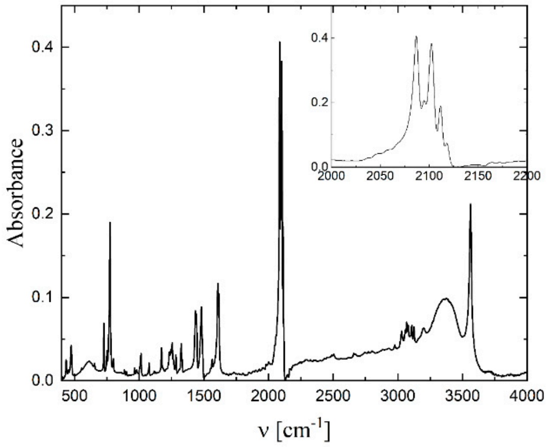

Figure S1. IR spectrum of **1**.

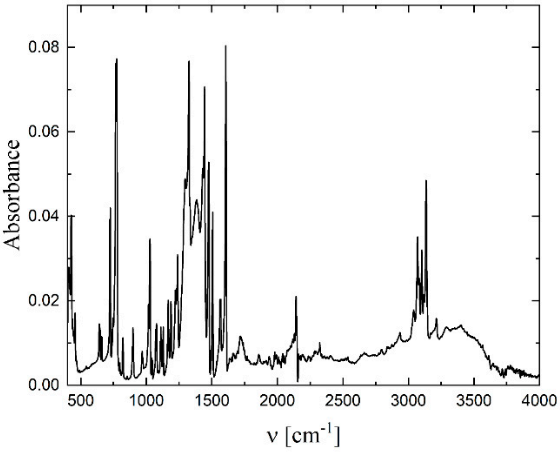

Figure S2. IR spectrum of **2**.

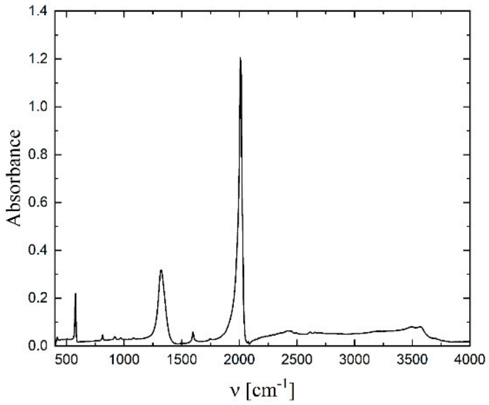

Figure S3. IR spectrum of **3**.

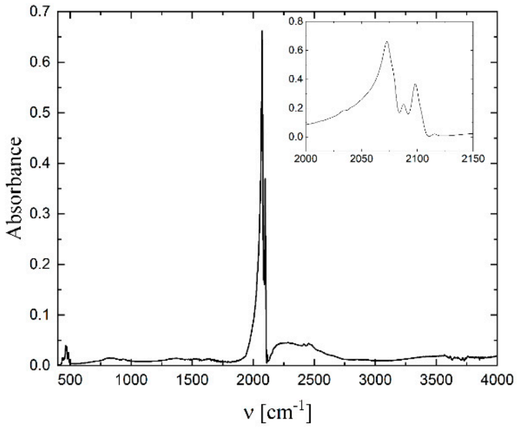

Figure S4. IR spectrum of **4**.

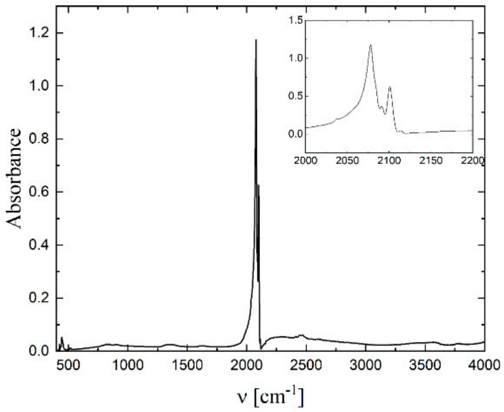

Figure S5. IR spectrum of **5**.

Table S1. Crystal data and structure refinement for **1-5**.

		**1**	**2**	**3**	**4**	**5**
Empirical formula		$C_{16}H_{10}N_8OTl_2W$	$C_{16}H_8N_8TlW$	$C_{72}Fe_{12}N_{81}O_{36}Tl_{57}$	$C_8N_8Tl_4W$	$C_8MoN_8Tl_4$
Formula weight		922.91	700.52	14894.82	1209.49	1121.58
Temperature [K]		281(2)	130(2)	293(2)	293(2)	293(2)
Wavelength [Å]		0.71073	1.54184	0.71073	0.71073	0.71073
Crystal system		Monoclinic	Monoclinic	Trigonal	Monoclinic	Monoclinic
Space group		P 2_1/n	P 2_1/n	R -3c	I 2/a	I 2/a
Unit cell dimensions	a [Å]	8.572(2)	7.59940(10)	18.9713(6)	16.0899(6)	16.0886(5)
	b [Å]	25.807(6)	14.7478(2)	18.9713(6)	7.3291(3)	7.3277(2)
	c [Å]	8.925(3)	15.4422(2)	30.2220(12)	26.4033(9)	26.4041(11)
	α [°]	90	90	90	90	90
	β [°]	105.38(3)	92.0430(10)	90	90.445(3)	90.502(3)
	γ [°]	90	90	120	90	90
Volume [$Å^3$]		1903.59(9)	1729.58(4)	9419.9(7)	3113.5(2)	3112.72(18)
Z		4	4	2	8	8
Density (calculated) [Mg/m^3]		3.220	2.690	5.251	5.161	4.787
Absorption coefficient [mm^{-1}]		22.942	29.956	49.494	48.627	42.059
F(000)		1624	1260	12432	4016	3760
Crystal size [mm^3]		0.10×0.10×0.05	0.10×0.10×0.10	0.05×0.05×0.05	0.30×0.02×0.02	0.05×0.05×0.05
Theta range for data collection [°]		2.588 to 28.605	4.146 to 75.299	2.967 to 30.360	2.884 to 30.631	2.885 to 30.682
Index ranges		$-11 \leq h \leq 11$<br>$-17 \leq k \leq 34$<br>$-11 \leq l \leq 10$	$-9 \leq h \leq 9$<br>$-18 \leq k \leq 18$<br>$-19 \leq l \leq 17$	$-21 \leq h \leq 19$<br>$-26 \leq k \leq 21$<br>$-38 \leq l \leq 37$	$-20 \leq h \leq 22$<br>$-10 \leq k \leq 9$<br>$-36 \leq l \leq 35$	$-21 \leq h \leq 19$<br>$-10 \leq k \leq 8$<br>$-33 \leq l \leq 32$
Reflections collected		8933	31587	13050	15734	15069
Independent reflections		4347 [R_{int} = 0.0349]	3525 [R_{int} = 0.1262]	2699 [R_{int} = 0.0475]	3959 [R_{int} = 0.0483]	3929 [R_{int} = 0.0700]
Completeness to theta [%]		99.9	99.6	99.7	99.9	99.9
Refinement method		Full-matrix least-squares on F^2	Full-matrix least-squares on F^2	Full-matrix least-squares on F^2	Full-matrix least-squares on F^2	Full-matrix least-squares on F^2
Data / restraints / parameters		4347/3/261	3225/0/235	2699/0/133	3959/0/190	3929/0/190
Goodness-of-fit on F2		1.023	1.130	1.056	1.011	1.064
Final R indices [I>2sigma(I)]		R_1 = 0.0331<br>wR_2 = 0.0586	R_1 = 0.0446<br>wR_2 = 0.1225	R_1 = 0.0425<br>wR_2 = 0.0904	R_1 = 0.0366<br>wR_2 = 0.0756	R_1 = 0.0575<br>wR_2 = 0.1544

R indices (all data)	R_1 = 0.0480 wR_2 = 0.0649	R_1 = 0.0448 wR_2 = 0.1227	R_1 = 0.0612 wR_2 = 0.0955	R_1 = 0.0498 wR_2 = 0.0787	R_1 = 0.0873 wR_2 = 0.1776
Largest diff. peak and hole [e·Å^{-3}]	1.882 and -2.390	3.061 and -2.614	1.842 and -2.987	2.250 and -4.571	2.925 and -3.289

Table S2. Selected bond lengths (Å) and angles (°) in **1-5**.

**1**		**2**		**3**		**4**		**5**	
Tl(1)-O(1)	2.778(6)	W(1)-C(13)	2.192(9)	Tl(2)-N(1)	2.774(10)	W(1)-C(1)	2.144(8)	Mo(1)-C(1)	2.144(16)
Tl(2)-O(1)	2.764(6)	W(1)-C(14)	2.146(9)	Tl(2)-N(3)	2.830(10)	W(1)-C(2)	2.174(8)	Mo(1)-C(2)	2.159(14)
W(1)-C(1)	2.141(7)	W(1)-C(15)	2.181(9)	Tl(3)-N(2)	2.833(11)	W(1)-C(3)	2.152(8)	Mo(1)-C(3)	2.165(16)
W(1)-C(2)	2.184(6)	W(1)-C(16)	2.136(8)	Fe(1)-C(4)	1.922(9)	W(1)-C(4)	2.129(9)	Mo(1)-C(4)	2.155(17)
W(1)-C(3)	2.160(7)	W(1)-C(17)	2.160(9)	Fe(1)-C(4)#1	1.922(9)	W(1)-C(5)	2.161(8)	Mo(1)-C(5)	2.193(18)
W(1)-C(4)	2.154(7)	W(1)-C(18)	2.178(9)	Fe(1)-C(4)#2	1.922(9)	W(1)-C(6)	2.153(8)	Mo(1)-C(6)	2.182(18)
W(1)-C(5)	2.151(8)	W(1)-N(1)	2.228(6)	Fe(1)-C(4)#3	1.922(9)	W(1)-C(7)	2.154(9)	Mo(1)-C(7)	2.188(18)
W(1)-C(6)#1	2.161(7)	W(1)-N(12)	2.231(6)	Fe(1)-C(4)#4	1.922(9)	W(1)-C(8)	2.179(8)	Mo(1)-C(8)	2.130(18)
W(1)-N(7)	2.211(5)	N(13)-C(13)	1.098(13)	Fe(1)-C(4)#5	1.922(9)	Tl(3)-N(1)	2.749(8)	Tl(1)-N(8)	2.750(17)
W(1)-N(18)	2.227(5)	N(14)-C(14)	1.143(12)	O(1)-N(5)	1.20(2)	Tl(3)-Tl(3)#1	3.6668(8)	Tl(1)-Tl(1)#1	3.6654(16)
N(1)-C(1)	1.157(8)	N(15)-C(15)	1.123(12)	O(2)-N(5)	1.173(15)	Tl(4)-N(4)#2	2.771(9)	Tl(2)-N(4)	2.751(16)
N(2)-C(2)	1.135(8)	N(16)-C(16)	1.162(11)	N(1)-C(1)	1.152(14)	Tl(2)-N(2)#3	2.785(9)	Tl(3)-N(7)#2	2.784(16)
N(3)-C(3)	1.149(8)	N(17)-C(17)	1.141(12)	N(4)-C(4)	1.140(14)	Tl(2)-Tl(1)#4	3.7348(5)	Tl(3)-Tl(4)	3.7319(11)
N(4)-C(4)	1.154(8)	N(18)-C(18)	1.124(13)	N(2)-C(2)	1.156(14)	Tl(1)-Tl(1)#5	3.7204(8)	Tl(4)-Tl(4)#3	3.7233(15)
N(5)-C(5)	1.150(9)	C(16)-W(1)-C(14)	101.6(3)	N(3)-C(3)	1.160(15)	N(1)-C(1)	1.162(11)	N(1)-C(1)	1.17(2)
N(6)-C(6)	1.139(8)	C(16)-W(1)-C(17)	79.0(3)	C(4)-Fe(1)-C(4)#1	91.9(4)	N(2)-C(2)	1.140(11)	N(2)-C(2)	1.13(2)
C(1)-W(1)-C(5)	108.2(3)	C(14)-W(1)-C(17)	140.5(3)	C(4)-Fe(1)-C(4)#2	88.1(4)	N(3)-C(3)	1.160(10)	N(3)-C(3)	1.12(2)
C(1)-W(1)-C(4)	142.4(2)	C(16)-W(1)-C(18)	72.6(3)	C(4)#1-Fe(1)-C(4)#2	180.0(8)	N(4)-C(4)	1.171(11)	N(4)-C(4)#4	1.16(2)
C(5)-W(1)-C(4)	80.3(3)	C(14)-W(1)-C(18)	69.7(3)	C(4)-Fe(1)-C(4)#3	91.9(4)	N(5)-C(5)	1.144(10)	N(5)-C(5)	1.12(2)
C(1)-W(1)-C(3)	80.2(3)	C(17)-W(1)-C(18)	73.1(3)	C(4)#1-Fe(1)-C(4)#3	88.1(4)	N(6)-C(6)	1.148(10)	N(6)-C(6)#2	1.13(2)

C(5)-W(1)-C(3)	135.9(2)	C(16)-W(1)-C(15)	71.2(3)	C(4)#2-Fe(1)-C(4)#3	91.9(4)	N(7)-C(7)	1.168(11)	N(7)-C(7)	1.12(2)
C(4)-W(1)-C(3)	69.9(2)	C(14)-W(1)-C(15)	74.2(3)	C(4)-Fe(1)-C(4)#4	88.1(4)	N(8)-C(8)	1.133(10)	N(8)-C(8)	1.18(2)
C(1)-W(1)-C(6)#1	71.0(2)	C(17)-W(1)-C(15)	139.1(3)	C(4)#1-Fe(1)-C(4)#4	91.9(4)	C(4)-W(1)-C(1)	146.0(3)	C(8)-Mo(1)-C(1)	71.2(7)
C(5)-W(1)-C(6)#1	70.1(3)	C(18)-W(1)-C(15)	121.2(3)	C(4)#2-Fe(1)-C(4)#4	88.1(4)	C(4)-W(1)-C(3)	76.0(3)	C(8)-Mo(1)-C(4)	146.4(8)
C(4)-W(1)-C(6)#1	78.4(2)	C(16)-W(1)-C(13)	142.4(3)	C(4)#3-Fe(1)-C(4)#4	180.0(7)	C(1)-W(1)-C(3)	82.3(3)	C(1)-Mo(1)-C(4)	138.2(6)
C(3)-W(1)-C(6)#1	72.5(2)	C(14)-W(1)-C(13)	82.1(3)	C(4)-Fe(1)-C(4)#5	180.0	C(4)-W(1)-C(6)	76.8(3)	C(8)-Mo(1)-C(2)	107.7(6)
C(1)-W(1)-C(2)	71.0(2)	C(17)-W(1)-C(13)	75.4(3)	C(4)#1-Fe(1)-C(4)#5	88.1(4)	C(1)-W(1)-C(6)	107.8(3)	C(1)-Mo(1)-C(2)	71.1(6)
C(5)-W(1)-C(2)	76.2(3)	C(18)-W(1)-C(13)	73.9(3)	C(4)#2-Fe(1)-C(4)#5	91.9(4)	C(3)-W(1)-C(6)	142.7(3)	C(4)-Mo(1)-C(2)	77.3(6)
C(4)-W(1)-C(2)	144.7(2)	C(15)-W(1)-C(13)	143.1(3)	C(4)#3-Fe(1)-C(4)#5	88.1(4)	C(4)-W(1)-C(7)	137.9(3)	C(8)-Mo(1)-C(3)	142.0(7)
C(3)-W(1)-C(2)	143.4(3)	C(16)-W(1)-N(1)	144.1(3)	C(4)#4-Fe(1)-C(4)#5	91.9(4)	C(1)-W(1)-C(7)	72.0(3)	C(1)-Mo(1)-C(3)	77.9(6)
C(6)#1-W(1)-C(2)	116.6(2)	C(14)-W(1)-N(1)	84.1(3)	C(4)-Fe(1)-C(4)#1	91.9(4)	C(3)-W(1)-C(7)	143.4(3)	C(4)-Mo(1)-C(3)	71.0(7)
C(1)-W(1)-N(7)	82.9(2)	C(17)-W(1)-N(1)	118.6(3)	C(4)-Fe(1)-C(4)#2	88.1(4)	C(6)-W(1)-C(7)	71.7(3)	C(2)-Mo(1)-C(3)	81.7(6)
C(5)-W(1)-N(7)	147.1(2)	C(18)-W(1)-N(1)	140.2(3)	C(4)#1-Fe(1)-C(4)#2	180.0(8)	C(4)-W(1)-C(5)	70.7(3)	C(8)-Mo(1)-C(6)	83.1(7)
C(4)-W(1)-N(7)	110.1(2)	C(15)-W(1)-N(1)	76.6(3)	C(4)-Fe(1)-C(4)#3	91.9(4)	C(1)-W(1)-C(5)	142.9(3)	C(1)-Mo(1)-C(6)	144.6(6)
C(3)-W(1)-N(7)	75.6(2)	C(13)-W(1)-N(1)	73.2(3)	C(4)#1-Fe(1)-C(4)#3	88.1(4)	C(3)-W(1)-C(5)	112.5(3)	C(4)-Mo(1)-C(6)	74.9(6)
C(6)#1-W(1)-N(7)	141.5(2)	C(16)-W(1)-N(12)	84.0(3)	C(4)#2-Fe(1)-C(4)#3	91.9(4)	C(6)-W(1)-C(5)	81.4(3)	C(2)-Mo(1)-C(6)	142.0(6)
C(2)-W(1)-N(7)	78.7(2)	C(14)-W(1)-N(12)	146.3(3)	C(4)-Fe(1)-C(4)#4	88.1(4)	C(7)-W(1)-C(5)	77.5(3)	C(3)-Mo(1)-C(6)	112.5(6)
C(1)-W(1)-N(18)	141.8(2)	C(17)-W(1)-N(12)	73.1(3)	C(4)#1-Fe(1)-C(4)#4	91.9(4)	C(4)-W(1)-C(2)	116.2(3)	C(8)-Mo(1)-C(7)	79.9(7)
C(5)-W(1)-N(18)	80.6(2)	C(18)-W(1)-N(12)	141.9(3)	C(4)#2-Fe(1)-C(4)#4	88.1(4)	C(1)-W(1)-C(2)	80.0(3)	C(1)-Mo(1)-C(7)	80.1(6)
C(4)-W(1)-	74.9(2)	C(15)-W(1)-	76.4(3)	C(4)#3-Fe(1)-	180.0(7)	C(3)-W(1)-	71.4(3)	C(4)-Mo(1)-	115.4(7)

N(18)		N(12)		C(4)#4		C(2)		C(7)	
C(3)-W(1)-N(18)	119.8(2)	C(13)-W(1)-N(12)	113.5(3)	C(4)-Fe(1)-C(4)#5	180.0	C(6)-W(1)-C(2)	144.8(3)	C(2)-Mo(1)-C(7)	145.3(6)
C(6)#1-W(1)-N(18)	143.2(2)	N(1)-W(1)-N(12)	73.2(2)	C(4)#1-Fe(1)-C(4)#5	88.1(4)	C(7)-W(1)-C(2)	78.8(3)	C(3)-Mo(1)-C(7)	73.4(6)
C(2)-W(1)-N(18)	75.6(2)			C(4)#2-Fe(1)-C(4)#5	91.9(4)	C(5)-W(1)-C(2)	73.6(3)	C(6)-Mo(1)-C(7)	71.5(6)
N(7)-W(1)-N(18)	72.7(2)			C(4)#3-Fe(1)-C(4)#5	88.1(4)	C(4)-W(1)-C(8)	77.6(3)	C(8)-Mo(1)-C(5)	72.2(6)
				C(4)#4-Fe(1)-C(4)#5	91.9(4)	C(1)-W(1)-C(8)	71.9(3)	C(1)-Mo(1)-C(5)	118.0(6)
						C(3)-W(1)-C(8)	76.1(3)	C(4)-Mo(1)-C(5)	77.5(7)
						C(6)-W(1)-C(8)	73.6(3)	C(2)-Mo(1)-C(5)	74.9(6)
						C(7)-W(1)-C(8)	117.8(3)	C(3)-Mo(1)-C(5)	144.2(6)
						C(5)-W(1)-C(8)	143.2(3)	C(6)-Mo(1)-C(5)	74.2(6)
						C(2)-W(1)-C(8)	139.3(3)	C(7)-Mo(1)-C(5)	137.8(7)
